# Meniere’s Disease

**Published:** 1911-12

**Authors:** 


### ABSTRACTS

Meniere's Disease. H. Bourgeois, *Le Progres Medical,* October 21, 1911. During the paroxysm, confinement to bed, darkness and quiet, blister or leeches to affected side, mustard foot baths, ether inhalations, ice compress to the epigastrium for nausea, chloroform water, etc.

Immediately after the paroxysm: a drastic cathartic, antisyphilitic measures if indicated—efficient in a very small proportion of cases; 606 is not advised in unilateral deafness but only in bad bilateral cases where there is everything to gain and nothing to lose.

Later treatment—after three or four weeks: Hypodermic injection of pilocarpine, ¼ centigram at a time, waiting ten minutes for salivation and sweating, and repeating if it does not occur till the limit of tolerance is reached—usually ½ centigrams. For children, the dose varies from 2 to 5 milligrams. Having established the tolerable dose, the injection is repeated daily for 12 to 24 days. If at the expiration of this time, the hearing is not improved, it is useless to continue. If the result is favorable, the course consists in 30 injections.

